# Whole abdominopelvic intensity-modulated radiation therapy for peritoneal disseminated rhabdomyosarcoma with three-year follow-up: a case report

**DOI:** 10.1186/s13014-019-1333-x

**Published:** 2019-07-15

**Authors:** Mariko Kawamura, Kuniyasu Okudaira, Yoshiyuki Itoh, Takeshi Kamomae, Eri Nishikawa, Hideki Muramatsu, Yoshiyuki Takahashi, Kazuki Yokota, Shinji Naganawa

**Affiliations:** 1 Department of Radiology, Nagoya University Hospital, 65 Tsurumai-cho, Showa-ku, Nagoya, 466-8560 Japan; 2 Department of Radiological Technology, Nagoya University Hospital, Nagoya, Japan; 3 Department of Therapeutic Radiology, Nagoya University Hospital, Nagoya, Japan; 4 Department of Pediatrics, Nagoya University Hospital, Nagoya, Japan; 5 Department of Pediatric Surgery, Nagoya University Hospital, Nagoya, Japan

**Keywords:** Whole abdominopelvic IMRT, Rhabdomyosarcoma, Peritoneal dissemination, Pediatric

## Abstract

**Background:**

The role of local radiotherapy in the treatment of metastatic rhabdomyosarcoma is important. However, with peritoneal dissemination, the application of local therapy is challenging. Although there are few reports addressing the efficacy of the whole abdominopelvic irradiation to peritoneal disseminated rhabdomyosarcoma patients, no precise curse of treatment nor the follow up result is explained in paper nor in the text.

**Case presentation:**

Six years old rhabdomyosarcoma boy with peritoneal dissemination was treated at our facility under COG D9803 protocol (vincristine, dactinomycin, and cyclophosphamide (VAC)). He underwent tumor resection on the 14th week according to the protocol. During surgery, the 2-cm residual tumor was completely resected, but in the pelvis, numerous nodules that were suspected as peritoneal disseminated tumors were observed. We administered 30 Gy/20fr whole abdominopelvic radiotherapy using volumetric modulated arc therapy (VMAT) technique and a 6 Gy sequential boost to pelvis after the surgery and completed the protocol treatment. During the course of treatment, the patient experienced G4 hematological toxicity and received multiple transfusions, particularly after whole abdominopelvic irradiation. He has achieved complete remission and is alive without evidence of recurrence and severe late adverse effect for 3 years. In terms of growth, his height and weight are within the average values for Japanese boys at the same age.

**Conclusion:**

By using the VMAT technique, a patient with peritoneal disseminated rhabdomyosarcoma can be treated, and a dose of 30 Gy to the whole abdominopelvis with concurrent chemotherapy may be tolerable.

## Background

The role of local therapy in the treatment of rhabdomyosarcoma is important, even for patients presenting with metastasis upon diagnosis [1]. However, when peritoneal dissemination is present, the application of local therapy is challenging. Although a few reports have described the efficacy and tolerability of abdominopelvic irradiation using intensity-modulated radiation therapy (IMRT) [2, 3], the precise course of treatment has not been identified. We present the case of a boy who had rhabdomyosarcoma with peritoneal dissemination and was treated with abdominopelvic irradiation using the IMRT technique with concurrent chemotherapy using the Children’s Oncology Group (COG) D9803 vincristine, dactinomycin, and cyclophosphamide (VAC) protocol [4]. He achieved complete remission and has survived for 3 years with no evidence of recurrence or severe late adverse effects. However, during the treatment, he experienced grade 4 (G4) hematotoxicity and received multiple transfusions, particularly after radiotherapy; thus, we believe that it is extremely important to share this experience.

## Case presentation

**Fig. 1:**
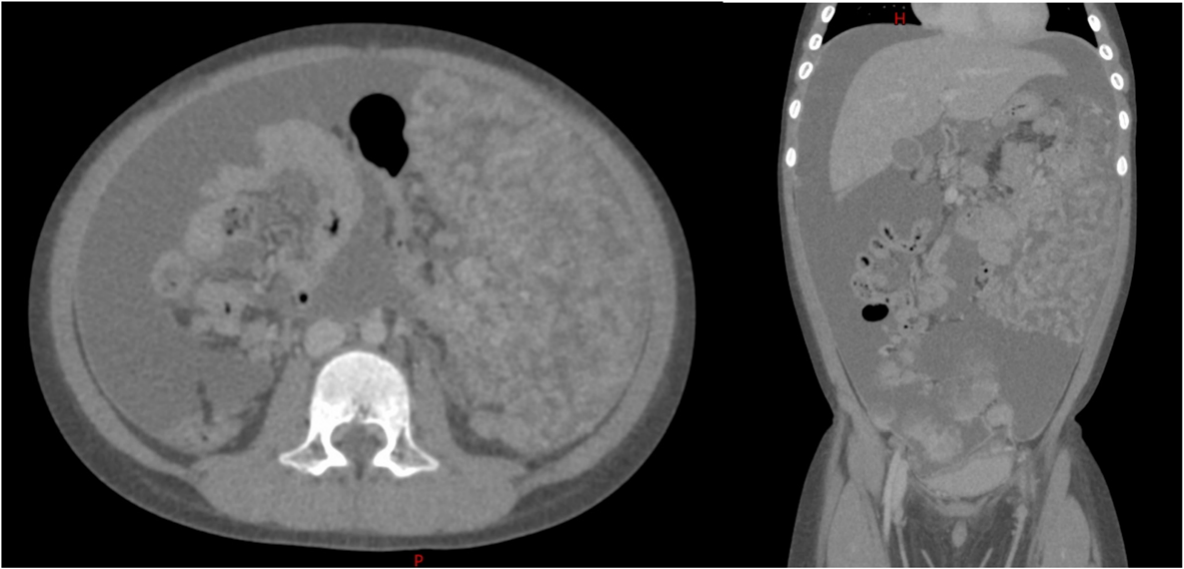
Computed tomography image obtained upon diagnosis showing multiple solid tumors in the pelvis, with a thickened omentum and ascites

**Fig. 2:**
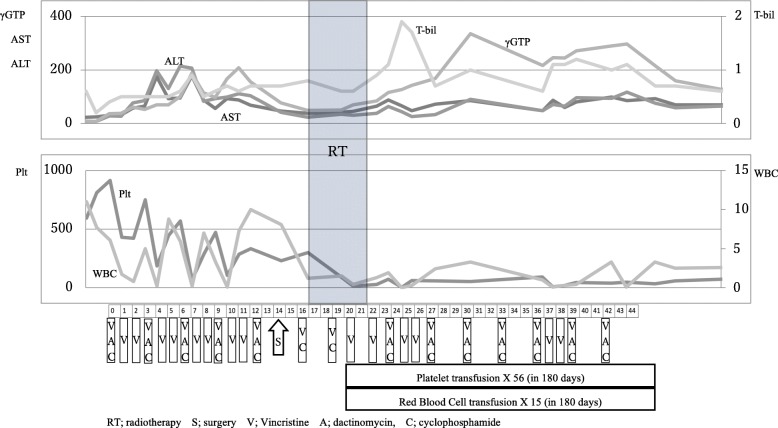
Therapeutic schedule and hematotoxicity. Platelet transfusion was performed 56 times within 180 days (every 2–4 days) after the completion of radiotherapy, and the patient withdrew from transfusion dependence 1 month after the last chemotherapy treatment

**Fig. 3:**
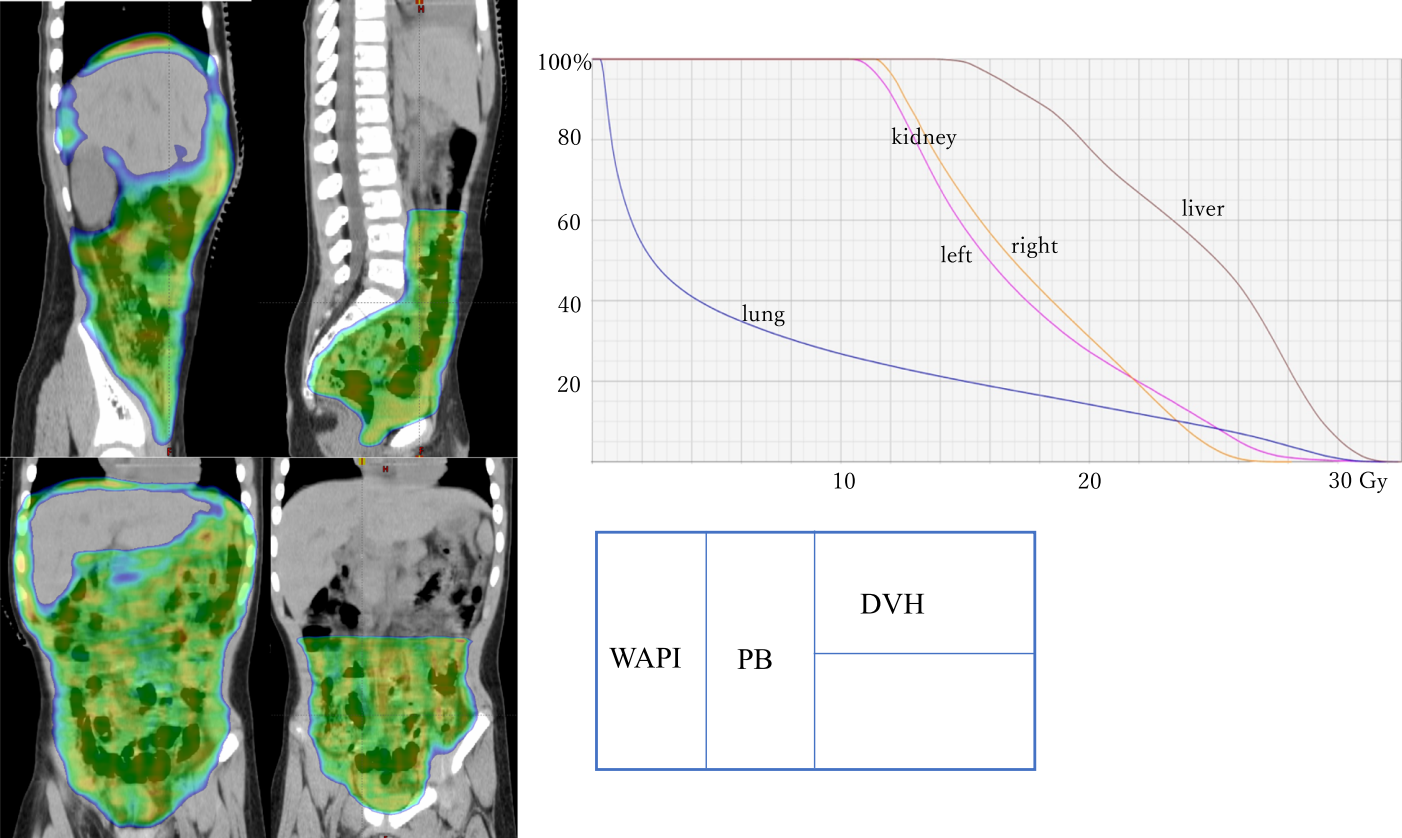
Intensity-modulated radiation therapy plan and dose-volume histogram (DVH): whole abdominopelvic irradiation (WAPI) of 30 Gy/20 fr and sequential pelvic boost (PB) of 6 Gy/4 fr (right). Images are color washed to > 90% dose with a global dose maximum < 109% of the dose for WAPI and < 108% of the dose for PB

**Fig. 4:**
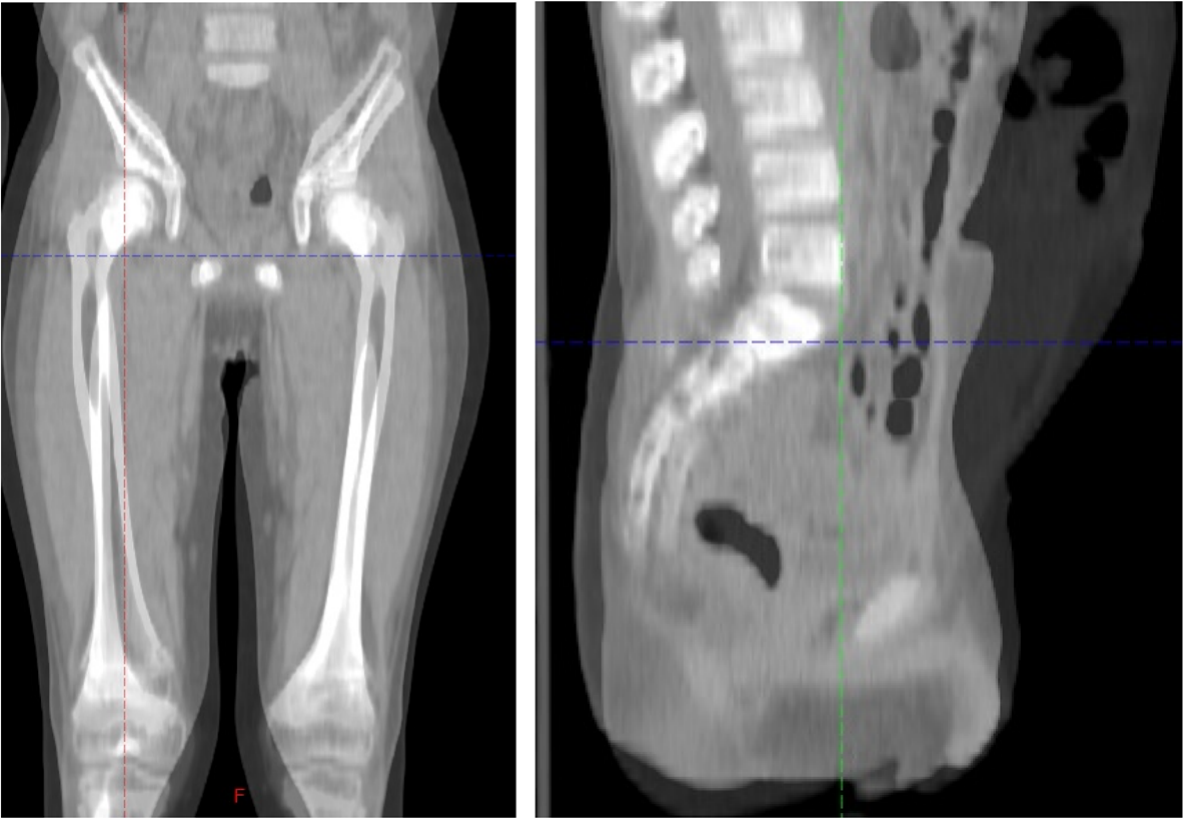
The fusion of images of the pelvis to the femur before treatment and 2 years after treatment. The volume of the left iliac bone increased from 66.1 to 81.1 cm^3^ (23% increase) and that of the femur increased from 154.7 to 202.5 cm^3^ (31% increase)

A 6-year-old boy presented to an emergency room with subacute abdominal fullness and acute abdominal pain. Computed tomography (CT) showed multiple solid tumors in his pelvis, with a thickened omentum and ascites (Fig. 1). Because a malignant tumor with peritoneal dissemination was suspected, he was remitted to our hospital. He underwent biopsy and was diagnosed with retroperitoneal rhabdomyosarcoma-embryonal type (positive for myogenin, myogenic differentiation 1, and desmin; and negative for α-smooth muscle actin and leukocyte common antigen). The patient was diagnosed with stage IV disease due to dissemination. However, considering that he was less than 10 years old and the histology demonstrated good prognosis, he was classified as an intermediate risk by IRS-V preoperative system. He was treated using the COG D9803 VAC protocol [4]. The schedule of chemotherapy, surgery, and radiotherapy is shown in Fig. 2. G4 hematotoxicity (leukopenia and thrombocytopenia) was observed (CTCAE version 5) after VAC, before the administration of radiotherapy, but had recovered quickly without delaying the chemo-schedule. He responded extremely well to the VAC, and after the first course of VAC, his ascites almost disappeared. By the 13th week, just before the surgery, CT showed a 2-cm residual tumor in his pelvis. He underwent tumor resection during the 14th week. However, innumerable nodules that were suspected to be peritoneal disseminated tumors were observed in the abdominopelvic region and resecting all of them was not feasible. The nodules were conspicuous in the pelvis; thus, the surgeon randomly picked three nodules for pathological diagnosis. Within the 2-cm tumor, observed via CT prior to the surgery, there was 5% residual tumor, but the tumor was resected without any marginal tumor cells. The three nodules that were suspected to be peritoneal dissemination and were resected for pathological analysis had no residual tumor cells. We concluded that the patient had achieved complete resection. However, there is a high chance of recurrence in the pelvis and abdomen. Therefore, we planned and delivered 30-Gy/20-fr whole abdominopelvic radiotherapy and a 6 Gy/4-fr sequential boost to the pelvis (Fig. 3) with 10-MV photons. For radiotherapy treatment planning, Eclipse version 13.6 (Varian Medical Systems, Palo Alto, CA) was used. The patient was placed in the supine position with vacuum fixation cushions and a thermoplastic body shell to immobilize his body and suppress breathing motions. He could not follow our instruction and hold his breath, thus CT was performed without holding the breath. The clinical target volume (CTV) for whole abdominopelvic radiotherapy consisted of the peritoneal cavity, excluding the kidney and liver and CTV for sequential pelvic boost consisted of the pelvic cavity caudal to the kidney. The planning target volume (PTV) was obtained by adding 5 mm to the CTV in all directions. The planning CT was obtained without holding breath, but we evaluated the dose distribution in both the inspiration and exhalation phases and calculated the CT dose in both phases to ensure a sufficient dose distribution for both phases. We adopted the volumetric modulated arc therapy (VMAT) technique for the initial and boost IMRT treatments plan. The dose-volume histogram (DVH) was as shown in Fig. 3. The dose covering 95% of liver, right kidney, and left kidney were 16Gy, 12Gy, and 12Gy. The percent of the right and left kidney volume receiving at lease 20Gy were 31 and 27%, respectively. Clinac ix (Varian Medical Systems, Palo Alto, CA) was used to carry out radiotherapy. We used kilovoltage X-ray imaging system first to apply the bone-match, then achieved cone beam CT image to check if the diaphragm was included in the PTV. During and after the radiotherapy, G1 dermatitis, G2 enteritis and cystitis, and G4 hematotoxicity (leukopenia and thrombocytopenia) were observed (CTCAE version 5). Leukopenia and thrombocytopenia were observed both before and after the radiotherapy, but was extremely severe, particularly after radiotherapy; as a result, the patient required multiple platelet transfusions (Fig. 2). Just after irradiation, the patient’s total bilirubin level transiently increased but eventually decreased to a normal level without any treatment. Transfusion dependence lasted for 1 month after the entire treatment was completed. However, eventually, the patient no longer required transfusion and was discharged. Considering the late adverse event, the patient experienced G1 cystitis and hepatic damage (CTCAE version 5), but did not experience kidney toxicity or radiation pneumonitis. He was in good health with no recurrence for 3 years. In terms of growth, his height increased from 121.7 to 132.4 cm, and his weight increased from 23.5 to 36.1 kg during 3 years of follow-up. The average height and weight for Japanese boys of his age are 119.6 ± 5.1 cm and 23.1 ± 4.1 kg before the treatment and 134.5 ± 5.8 cm and 31.9 ± 7 kg after 3 years; thus, his height and weight are within the 1SD for Japanese boys of the same age. The fusion of images of the pelvis to the femur before treatment and 2 years after treatment is shown in Fig. 4. The volume of his left iliac bone, which was within the irradiating field, increased from 66.1 to 81.1 cm^3^ (23% increase), whereas that of the femur, which was outside of the irradiating field, increased from 154.7 to 202.5 cm^3^ (31% increase).

## Discussion

Mohan et al. have reported an extremely interesting and enlightening result regarding stage IV rhabdomyosarcoma; that is, in patients who did not undergo radiotherapy, the positive outcome may be limited [1]. In addition, Casey et al. have reported the efficacy of whole abdominopelvic radiation therapy in patients with peritoneal dissemination [2]. However, the cases were extremely limited, and effectiveness and safety in terms of irradiating dose have not been validated. Furthermore, whether the use of IMRT in the pelvic bone may deform the pelvic bone of pediatric patients, who are in a period of growth, has not been verified.

We believe that this case study illustrates two important points. First, a patient with rhabdomyosarcoma and peritoneal dissemination can be treated using the IMRT technique, and a dose of 30-Gy in 20 fractions (1.5 Gy/fr) to the whole abdominopelvic area with concurrent chemotherapy may be tolerable. However, pediatric oncologists should be aware that treatment of pediatric patients with this method, whole abdominopelvic IMRT with concurrent chemotherapy, may result in severe hematological toxicity that may last throughout the treatment course. Because we do not irradiate the whole body, particularly the sternum, we believe that patients will eventually achieve transfusion independence. However, because the treatment course is extremely long, it may be difficult to keep the condition of a patient with severe hematological toxicity stable. Therefore, this treatment is not recommended if the pediatric oncologist is not confident about treating pediatric patients with severe hematological toxicity for more than 3 months.

Second, IMRT to the pelvic bone did not cause deformation at 3 years of follow-up. A total dose of ≥10 Gy causes stature loss in pediatric patients [5], and to avoid the risk of spinal curvature, radiation oncologists do not irradiate the bones of pediatric patients in an asymmetric field. Because we used the VMAT technique, the dose to the pelvic bone was heterogeneous. Therefore, our concern was that the retardation in pelvic bone growth may be asymmetric. In our patient, the pelvic bone grew without deformation. We believe that the use of the VMAT technique may reduce the risk of asymmetric growth of the irradiated bone by obscuring the boundary of the high and low dose to the pelvic bone. We are not certain if the difference in iliac and femur volume increase (21% vs 31% increase) were caused by irradiation, or if it is because of normal growth in a boy of 6–9 years of age. Therefore, a careful follow-up for a longer period is required to completely assess this issue.

Although a 3-year follow-up is not sufficient for discussing the curability of this disease, a patient who is not cured usually experiences recurrence within 2 years; thus, we believe that the treatment course in this patient was favorable. Moreover, he must be continuously followed up to fully assess late toxicity and curability. However, reports on the precise course that a patient may undergo after whole abdominopelvic irradiation are valuable. We recommend this method only to institutions that are highly experienced in treating severe hematologic toxicity. However, as long as there is enough unirradiated bone marrow, a patient will eventually achieve transfusion independence.

## Conclusion

The treatment of stage IV rhabdomyosarcoma is extremely challenging. However, whole abdominopelvic IMRT can be safely performed to peritoneal disseminated patients, and this technological improvement may increase the chance of a good outcome with close cooperation between surgeons and oncologists.

## Data Availability

Data sharing is not applicable to this article as no datasets were generated or analyzed during the current study.

## Abbreviations

COG: Children’s Oncology Group
CT: Computed Tomography
CTCAE: Common terminology criteria for adverse events
IMRT: Intensity modulated radiation therapy
PB: Pelvic boost
VMAT: Volumetric modulated arc therapy
WAPI: whole abdominopelvic irradiation
